# Trichinellosis outbreak due to wild boar meat consumption in southern Italy

**DOI:** 10.1186/s13071-017-2052-5

**Published:** 2017-02-28

**Authors:** Iulia Adelina Turiac, Maria Giovanna Cappelli, Rita Olivieri, Raffaele Angelillis, Domenico Martinelli, Rosa Prato, Francesca Fortunato

**Affiliations:** 1 European Programme for Intervention Epidemiology Training (EPIET), European Centre for Disease Prevention and Control, (ECDC), Stockholm, Sweden; 2 Department of Medical and Surgical Sciences, University of Foggia, Foggia, Italy; 3 Foggia Local Health Unit, Manfredonia, Italy

**Keywords:** *Trichinella britovi*, Trichinellosis, Italy, Wild boar meat, Zoonosis

## Abstract

We report a *Trichinella britovi* outbreak investigated during February-March 2016 in southern Italy. The source of infection was meat from infected wild boars that were illegally hunted and, hence, not submitted to post-mortem veterinary inspection. Thirty persons reported having eaten raw dried homemade sausages; five cases of trichinellosis were confirmed. Wild game meat consumers need to be educated about the risk for trichinellosis.

## Letter to the Editor

In the European countries, the wildlife and domestic reservoirs of *Trichinella* spp*.* still pose a risk for humans, leading to outbreaks. Wild carnivore mammals are of particular importance since a large number of hunted animals escape veterinary control [1]. According to the epidemiological data the European Centre for Disease Prevention and Control (ECDC), trichinellosis is most prevalent in eastern Europe but also in Italy and Spain where outbreaks were reported in the past 10 years [2]. In Italy, from 1985 to 2016, *Trichinella* spp. were detected in 354 animals (both domestic and wild); 97.5% of these were identified as *Trichinella britovi*, 2.2% as *Trichinella pseudospiralis*, and 0.3% as *Trichinella spiralis* at the European Union Reference Laboratory for Parasites (EURL) [3]. Cultural traditional habits of eating raw or undercooked meat play a key role in the spread of the disease and were responsible for past outbreaks [4].

We report an outbreak investigation conducted in the city of Manfredonia, Apulia Region, located in the Gargano National Park, where currently only the sylvatic cycle of *Trichinella* spp. [4] is present and poaching is a common practice. The source of infection was meat from two infected wild boars illegally hunted on 12 December 2015, hence not sampled for post-mortem examination for the presence of *Trichinella*, as per the EU legislation, and consumed as raw dried homemade sausages.

A 36 year-old hunter was admitted to the “Casa Sollievo della Sofferenza” Hospital, San Giovanni Rotondo city on 25 January 2016, suffering from fever (temperature 40–41 °C), myalgia, facial and periorbital swelling, diarrhoea, vomiting, abdominal pain and night sweating. These symptoms were developed 20 days before hospitalisation. Laboratory analysis showed marked eosinophilia (42%) and increased CPK (472 UI/l). The patient reported that his wife and son had similar symptoms after having eaten together wild boar meat derived from the same hunting trip (symptoms onset on 4 and 7 January, respectively). All three suspected cases were reported to the local public health authority, responsible for investigating the source of infection and limiting its spread.

On 28 January 2016, a diagnosis of trichinellosis was confirmed for the father and son by enzyme-linked immunosorbent assay (ELISA) on the patients’ serum specimens collected and sent to the EURL for parasites.

Between February and March 2016, an epidemiological investigation started to search actively the potentially exposed persons, using a questionnaire to gather information and trace-back the distribution of the suspected meat. Despite the initial hesitancy, due to the illegal behaviour, the index case provided a list of the consumers of the wild boar meat. The veterinary services obtained two samples of two different leftover sausages for laboratory examinations. *Trichinella* spp. larvae were detected using the magnetic stirrer artificial digestion method, which revealed 15 and 17 larvae/g of meat, respectively. *Trichinella britovi* was identified using the multiplex PCR method at the EURL for parasites.

A confirmed case was defined as a person who had consumed wild boar meat and/or meat products, had presented at least three of the following symptoms: fever, muscle soreness and pain, diarrhoea, facial oedema, eosinophilia, subconjunctival, subungual and retinal haemorrhages, and ELISA seropositivity.

Thirty persons reported having eaten raw dried sausages between 20 and 31 December 2015. A total of five cases were confirmed (including two 10-year-old children) in two different family clusters (Cluster 1: the index case, his wife and son; Cluster 2: a second hunter and his son) with symptoms onset between 4 and 15 January 2016, 15–21 days after eating the sausages. Four of the five cases had an ELISA positive result at the first time point (2–4 weeks after the onset of symptoms), while in the index case’s wife the seroconversion occurred on 17 March 2016, at the second time point (6–8 weeks after the first time point). No other case than the index required hospitalization. All cases received anthelmintic treatment (mebendazole) within 38 days on average (range: 36–39) between exposure and treatment start, and had an uneventful recovery.

Compared to the cases who reported the consumption of sausages several times, the 25 non-cases ate them on a single occasion, suggesting a dose-response relationship. Serology was also performed on 22 out of 25 of non-cases (three refused testing): none of those tests were positive. Three non-cases had an eosinophilia level above the normal limits (range: 7.8–11.7%).

The long incubation period and the delay of seroconversion made the rapid response to this *Trichinella* outbreak difficult, as also highlighted in a recent article by Messiaen et al. [2]. Moreover, it is possible that we missed some additional cases with milder or no symptoms, not seeking medical attention, due to the reluctance of the people involved in providing more information. However, the combination of coordinate actions and communication among different authorities (hospital, local health unit, veterinary services) was essential in order to determine the extent of the outbreak, to identify its source and to implement control measures.

As the illegal nature of poaching makes veterinary control impossible, the cultural habit of consuming raw or undercooked meat continues to be the primary risk factor for acquiring trichinellosis. Hunters and wild game meat consumers need to be educated about the risk for trichinellosis and the importance of proper handling and cooking game meat. In addition, it is necessary continually raising the awareness on the epidemiological and clinical features of this zoonosis among healthcare personnel for an immediately suspicion of the disease.

## References

[1] Fichi G, Stefanelli S, Pagani A, Luchi S, De Gennaro M, Gómez-Morales MA, et al. Trichinellosis outbreak caused by meat from a wild boar hunted in an Italian region considered to be at negligible risk for *Trichinella*. Zoonosis Public Health. 2015;62(4):285–91. https://www.ncbi.nlm.nih.gov/pubmed/25103623. 10.1111/zph.12148 25103623

[2] Messiaen P, Forier A, Vanderschueren S, Theunissen C, Nijs J, Van Esbroeck M (2016). Outbreak of trichinellosis related to eating imported wild boar meat, Belgium, 2014. Euro Surveill..

[3] Pozio E (2016). *Trichinella* in Italy and in European union.

[4] Pozio E, La Rosa G, Gomez Morales MA (2001). Epidemiology of human and animal trichinellosis in Italy since its discovery in 1887. Parasite..

